# Supplementary material for: Molecular basis of TASL recruitment by the peptide/histidine transporter 1, PHT1
Source: Nat Commun. 2023 Sep 14;14:5696. doi: 10.1038/s41467-023-41420-5 (PMC10502012; doi:10.1038/s41467-023-41420-5)
Supplement: Supplementary file 1 — Supplementary information [file 41467_2023_41420_MOESM1_ESM.pdf]

SUPPLEMENTARY MATERIAL

Molecular basis of TASL recruitment by the Peptide/Histidine Transporter 1, PHT1

Tânia F. Custódio[1,2], Maxime Killer[1,2,3], Dingquan Yu[1,2,3], Virginia Puente[1,2], Daniel P. Teufel[4], Alexander Pautsch[4], Gisela Schnapp[4], Marc Grundl[4], Jan Kosinski[1,2,5] and Christian Löw[1,2#]

[1] Centre for Structural Systems Biology (CSSB), Notkestraße 85, 22607 Hamburg, Germany.

[2] European Molecular Biology Laboratory (EMBL) Hamburg, Notkestraße 85, 22607 Hamburg, Germany.

[3] Collaboration for joint PhD degree between EMBL and Heidelberg University, Faculty of Biosciences.

[4] Boehringer Ingelheim Pharma, Birkendorferstr 65, 88397 Biberach, Germany.

[5] Structural and Computational Biology Unit, European Molecular Biology Laboratory, Heidelberg, Germany.

[#] Corresponding author

Christian Löw

European Molecular Biology Laboratory Hamburg, Notkestrasse 85, D-22607 Hamburg, Germany.

Phone: +49 40 8998 87570

e-mail: christian.loew@embl-hamburg.de

Twitter handle: @AllUNeedIsLoew

Supplementary Fig. 1

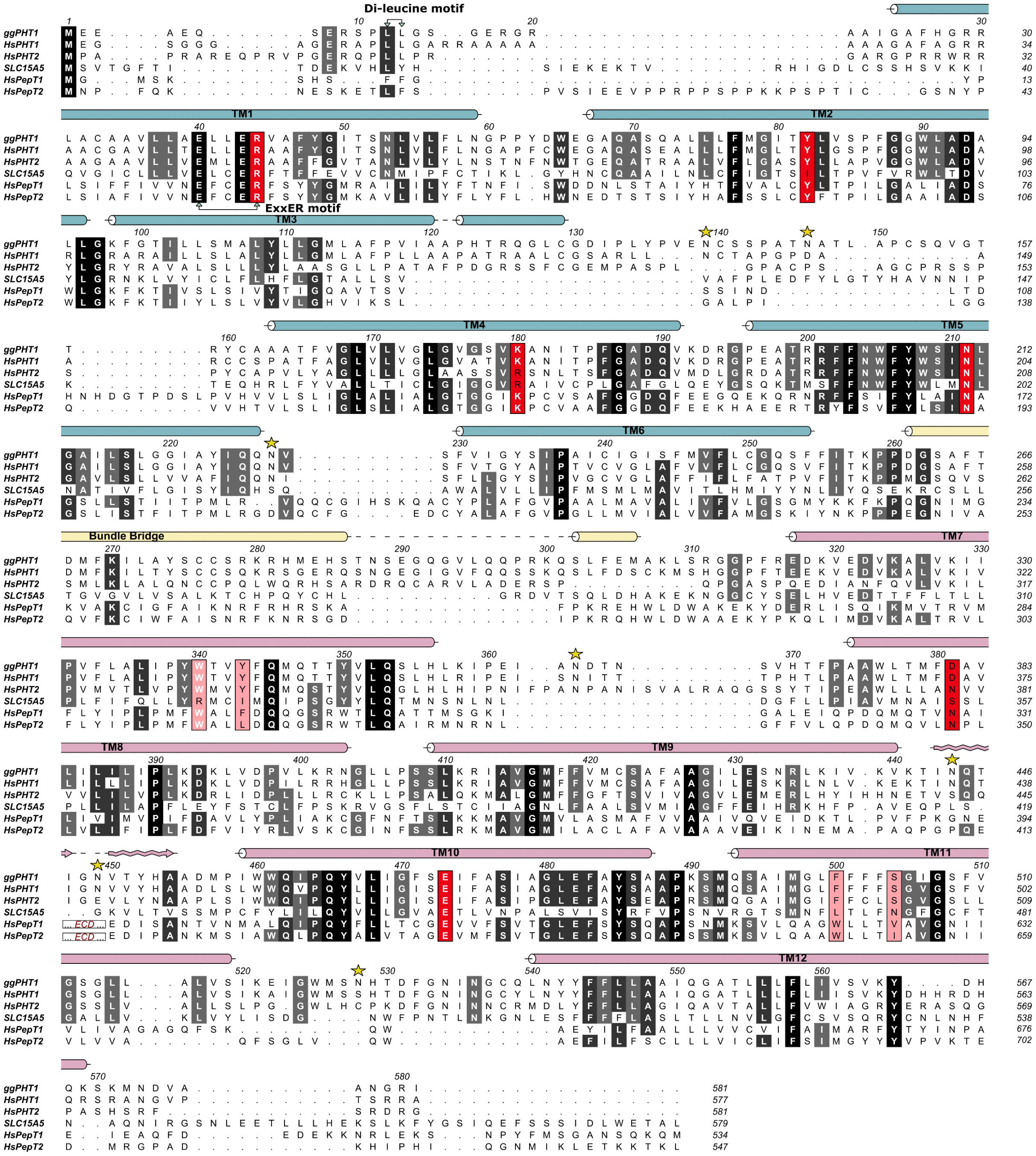

Supplementary Figure 1 - Multiple sequence alignment of chicken PHT1 with other human POT family members. Alignment between chicken PHT1 (accession number F1NG54), human PHT1 (accession number Q8N697), human PHT2 (accession number Q8IY34), human SLC15A5 (accession number A6NIM6), human Pept1 (accession number P46059) and human PepT2 (accession number Q16348). Conserved residues are highlighted with grey-scale, where black is perfectly conserved. Colored tubes represent α-helices found in the N-domain (blue), bundle bridge (yellow) and C-domain (pink). Residues highlighted in

red are part of the peptide binding site, coordinating the N- and C-termini of peptides, while residues highlighted in salmon are part of the side-chain coordination of peptides.

Supplementary Fig. 2

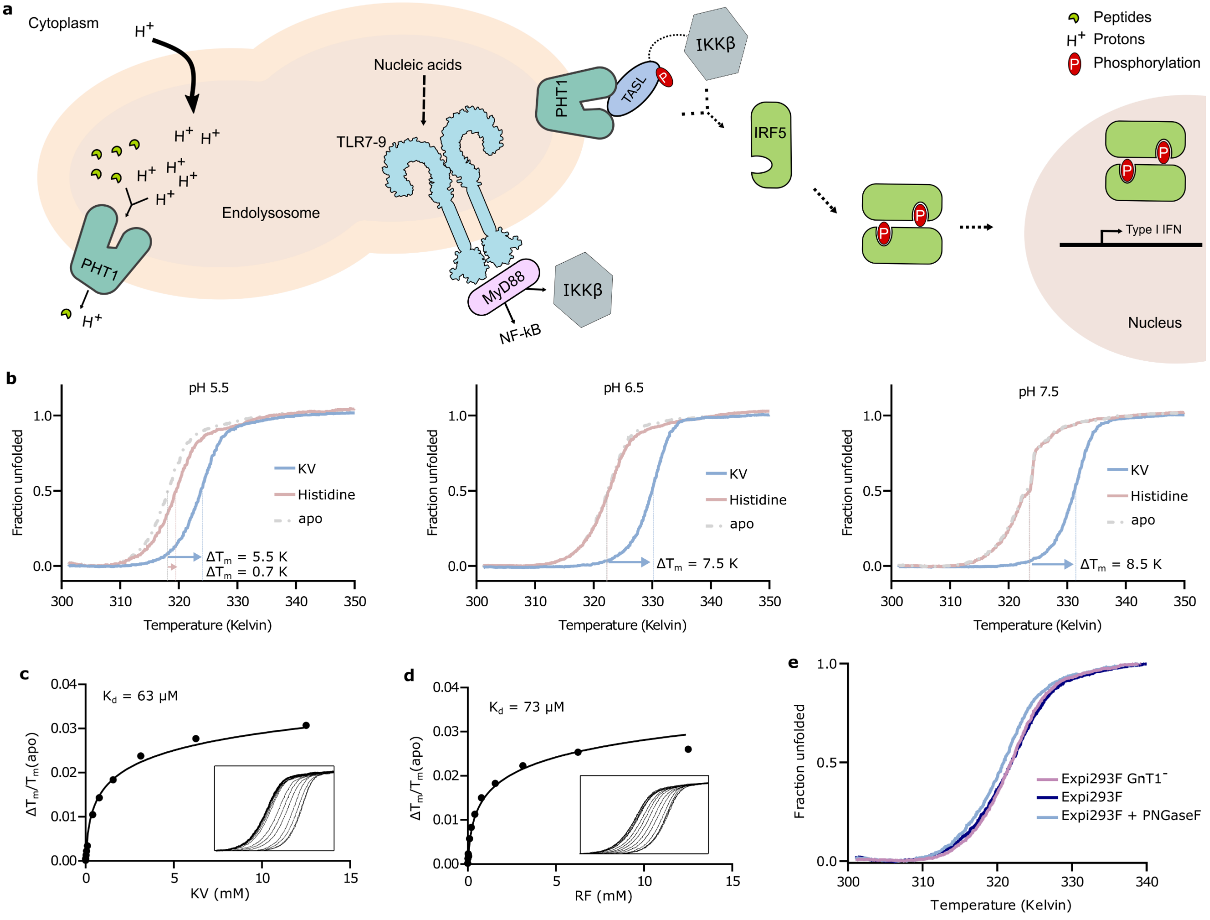

Supplementary Figure 2 – Functional characterization of PHT1 by thermal stability assays. a) Schematic representation of PHT1-TASL position in the TLR signaling pathway. PHT1 uses the proton gradient as the motive force for the transport of histidine or oligopeptides from the inside of the lysosome to the cytosol, thereby regulating endosomal pH. Endolysosomal TLRs (TLR7-9) recognize nucleic acids derived from foreign pathogens and in response recruit the universal adaptor myeloid differentiation primary-response protein 88 (MyD88). The activation of MyD88 signaling leads to the transcription of pro- inflammatory cytokines via the nuclear factor-κB (NF-κB). In addition, PHT1 forms a complex with the adaptor protein TASL which becomes phosphorylated by the IKKβ kinase and in turn can recruit and activate IRF5 and subsequently promote the production of type I IFN genes. This figure was incited by the work of Heinz *et al.* [1]. b) pH dependent screen of the thermal stability of PHT1 alone or in the presence of L-Histidine and the Lysine-Valine (KV) dipeptide. Normalized unfolding transitions from the $F_{350:330}$ signal are shown. Changes in the melting temperature of PHT1 in the presence of Histidine or KV dipeptide as compared to the melting temperature of PHT1 alone (ΔT_m) are annotated. Thermal unfolding titration of c) KV

dipeptide or d) Arginine-Phenylalanine (RF) dipeptide with PHT1. Data extrapolated from the $F_{350:330}$ signal was fitted following a modified Hall's approach and normalized unfolding transitions are shown. e) Thermal unfolding transitions of different glycosylation forms of PHT1. Source data for relevant information are provided as a Source Data file.

Supplementary Fig. 3

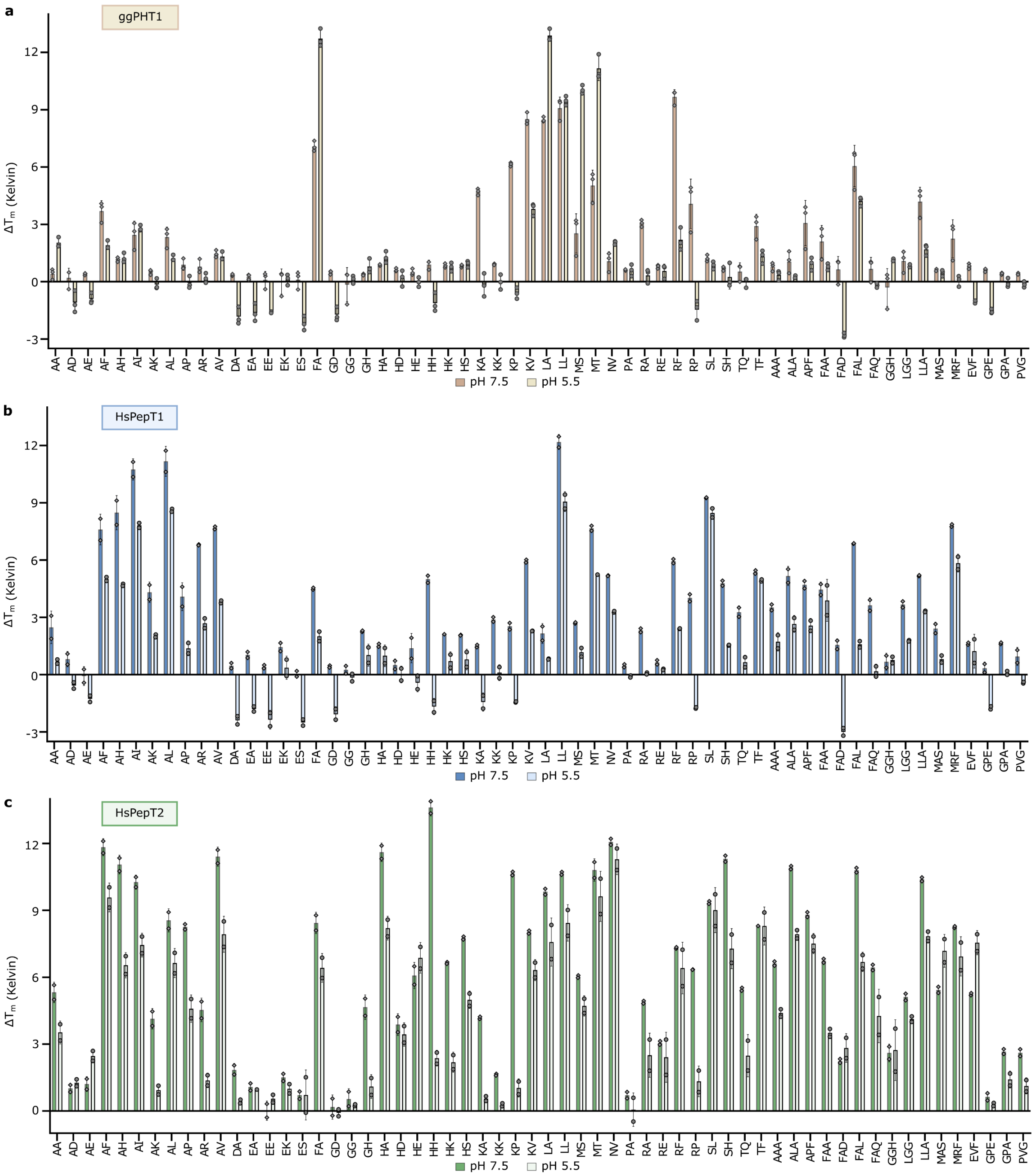

Supplementary Figure 3 – Substrate preference of PHT1 versus PepT1 and PepT2. a) Thermal stability changes of a) PHT1 b) PepT1 and c) PepT2 screened with a ligand library at a concentration of 5 mM at pH 7.5 and pH 5.5. Changes in the melting temperature of the protein in the presence of a ligand compared to the melting temperature of the protein alone (ΔT_m) are shown. Library is noted using the 1 letter abbreviation for amino acids. Data represent the mean ± SD of two or more technical replicates. Binding of histidine containing

peptides is detected for PepT1 and PepT2 but not for PHT1. Source data for relevant information are provided as a Source Data file.

Supplementary Fig. 4

a

	Enrichment Phage Display 2	Enrichment Phage Display 3	Number of ELISA hits (total analysed)	Number of unique binders (total sequenced)	Well behaved binders (total analysed)
Concave	2.2	2.8	87/94	1 (55)	0 (1)
Loop	5.0	5.1	85/94	32 (53)	18 (32)
Convex	5.8	3.7	84/94	8 (46)	2 (8)

* During the selection the cocave library was cross-contaminated.

b

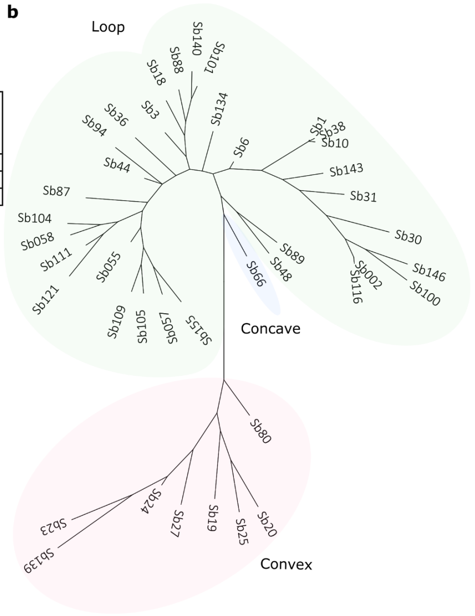

c

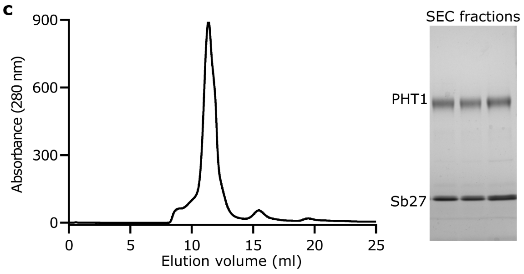

Supplementary Figure 4 - Sybody selections against PHT1. a) Sybody selection statistics for the three different libraries. b) Radial phylogenetic tree of all unique binders identified in this study. Sequence alignment was performed using PROMAL3D and the phylogenetic tree was constructed using the maximum likelihood (ML) analysis in MEGA. c) Gel-filtration chromatography using a Superdex 200 10/300 column and SDS-PAGE analysis of PHT1 in complex with Sb27. Source data for relevant information are provided as a Source Data file.

Supplementary Fig. 5

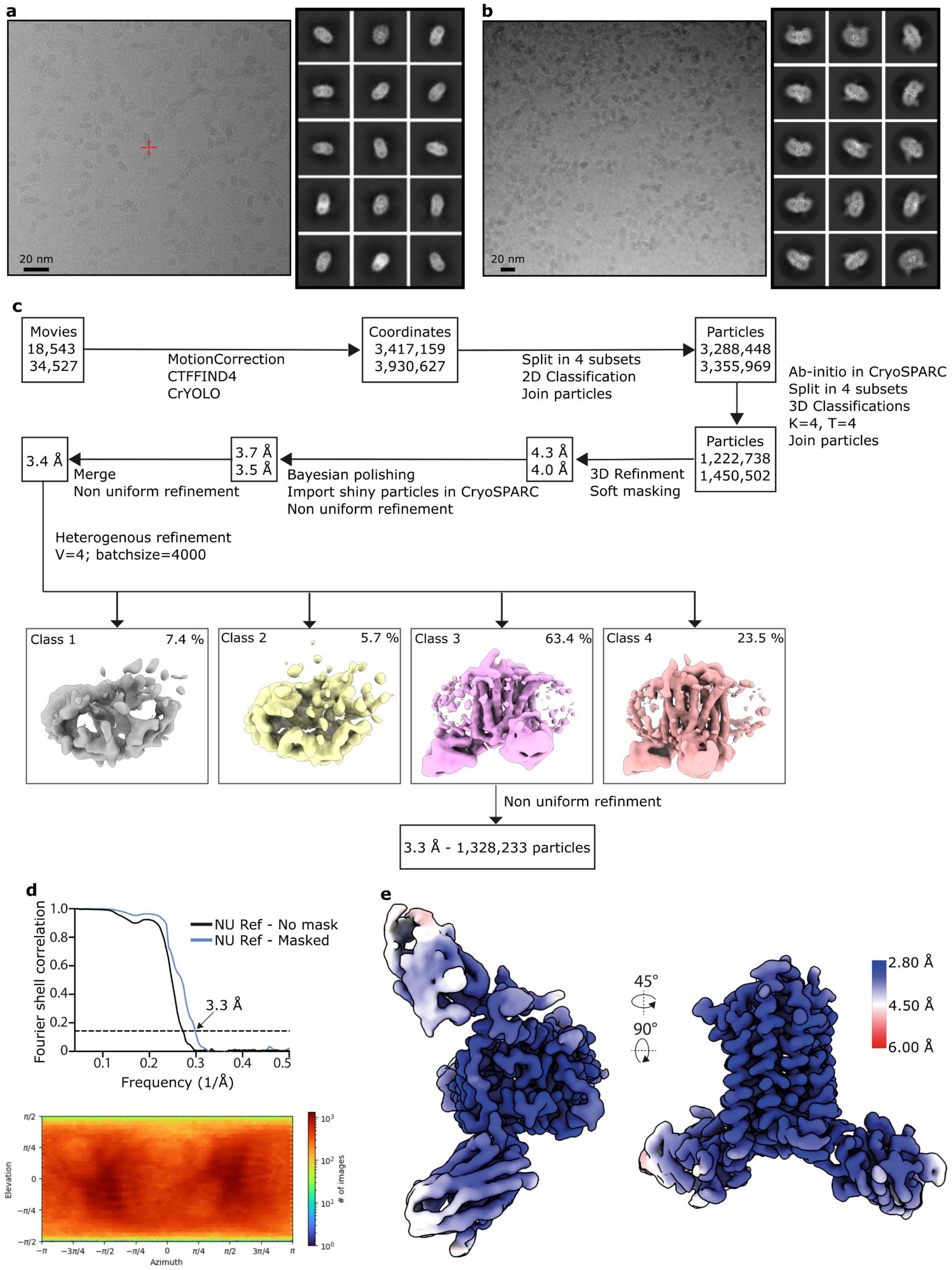

Supplementary Figure 5 – Cryo-EM data collection and processing of PHT1 in complex with Sb27. Side-by-side comparison of PHT1 samples imaged by cryo-EM. a) PHT1

transporter alone. b) PHT1 in complex with Sb27. c) Summary of the data processing workflow. d) The Fourier transforms over different shells on frequency space, of two independent volumes (half maps) were compared (FSC) and plotted as a function of spatial frequency, to estimate the overall resolution using the 0.143 cut-off threshold (top). Angular distribution of particles used in the final reconstruction is shown (bottom). e) 3D volume, coloured by local resolution, estimated in cryoSPARCv3 using 0.5 as FSC threshold.

Supplementary Fig. 6

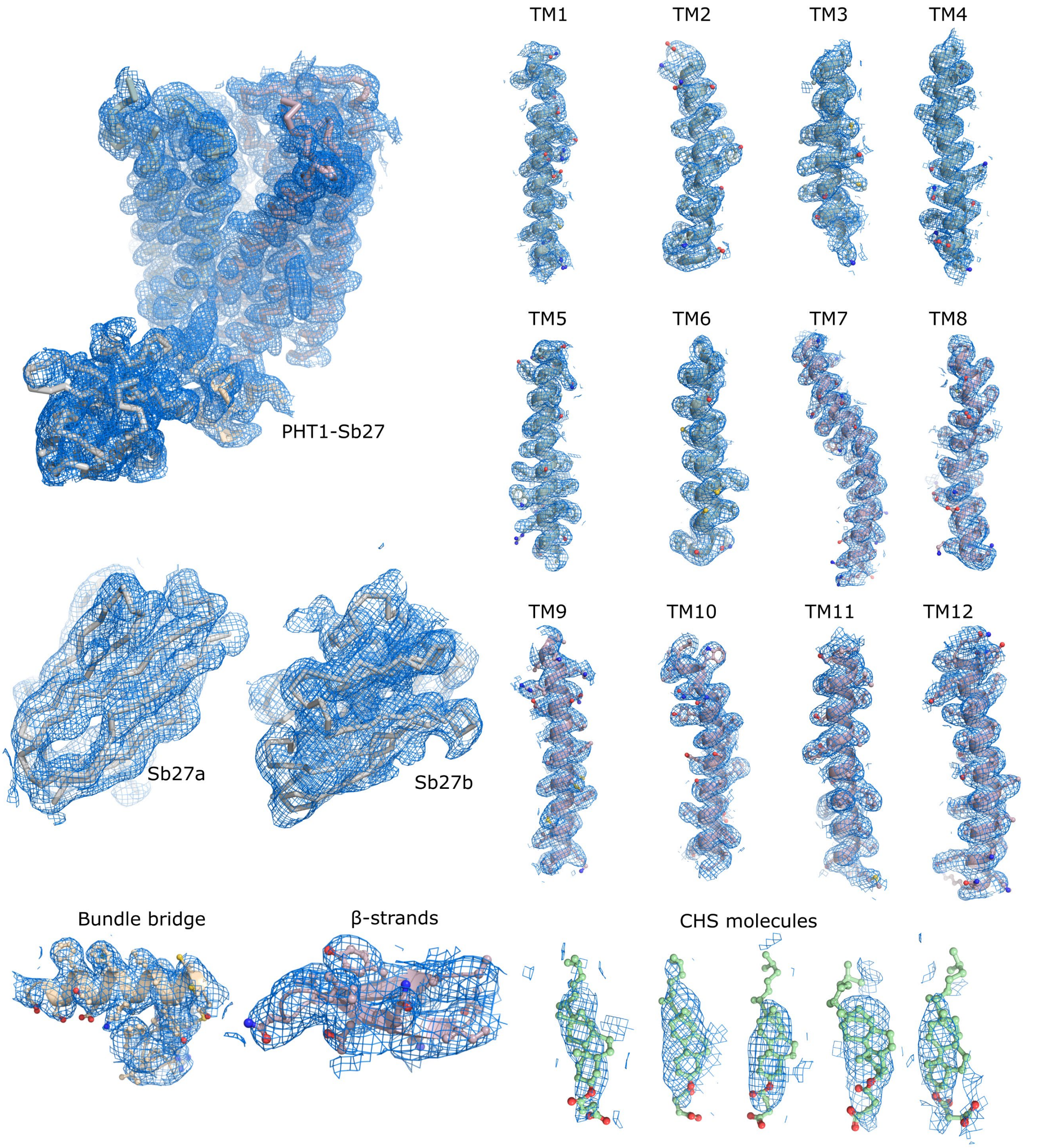

Supplementary Figure 6 - Density map of the 3.3 Å cryo-EM structure of PHT1 in complex with Sb27. The density map is shown as blue mesh and depicts a density within a 2.0 Å radius of any modelled atom. The final model is overlaid and amino acid side chains are shown as sticks. Maps are shown for individual sybodies, helices, domains and ligands.

Supplementary Fig. 7

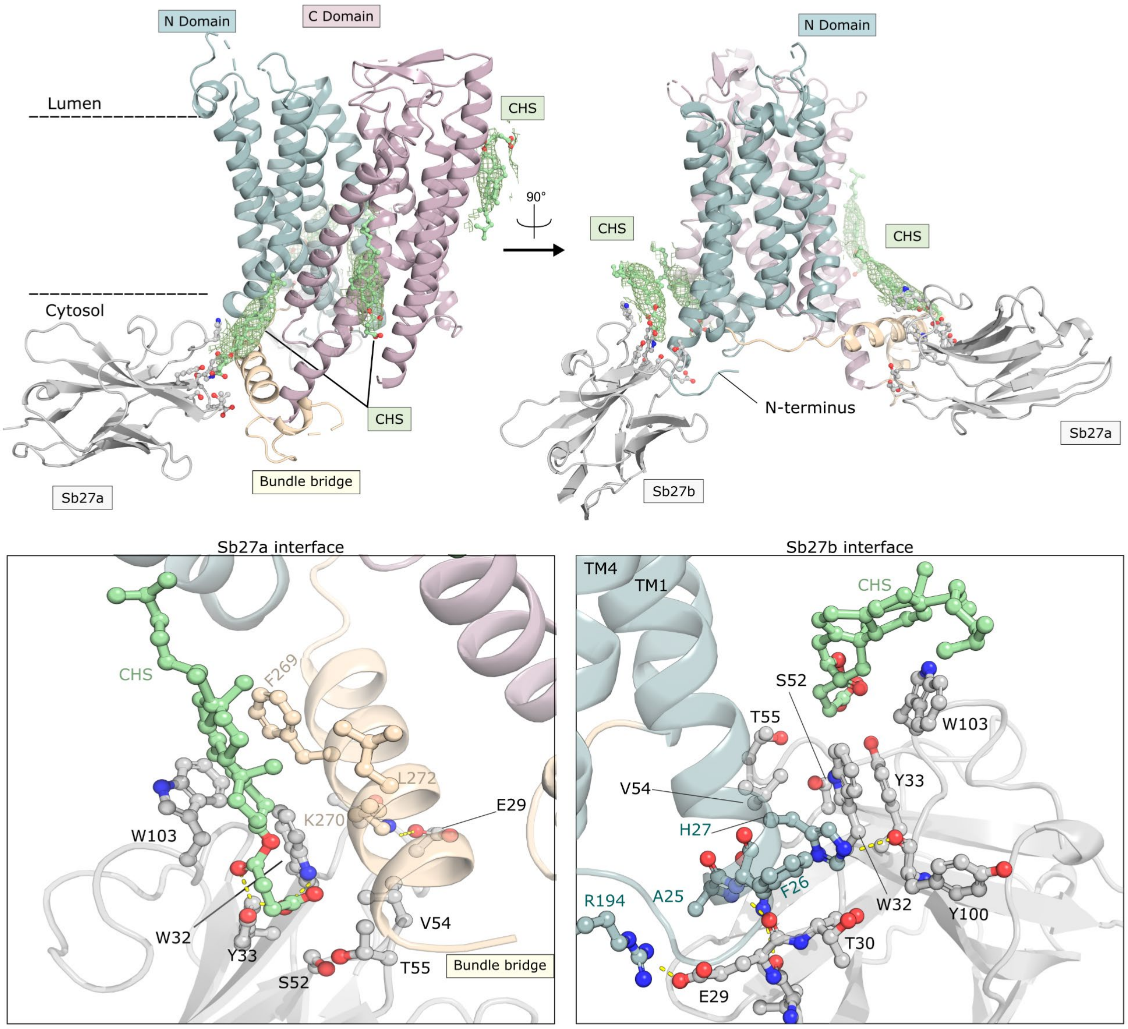

Supplementary Figure 7 – Binding interface between PHT1 and Sb27. 3D reconstruction identified two copies of Sb27 on the cytosolic side of PHT1. One of the epitopes is formed by the N-terminal residues preceding TM1 and the cytosolic interface of TM1 and TM4 (Sb27b interface), while the other epitope is defined by the bundle bridge (Sb27a interface). In both cases a CHS molecule contributes to the interaction between Sb27 and PHT1 epitopes. The cholesterol moiety is sandwiched between the transporter and Trp103 from the CDR3 loop of Sb27. The succinic acid moiety penetrates a pocket formed by polar contacts. In case of the Sb27a interface, Sb27 Glu29 is in close proximity to Lys271 of PHT1, while the same Glu29 residues of Sb27 forms a salt bridge with Arg94 of PHT1 in the other interface. These data explain how the same sybody can recognize two different conformational epitopes on PHT1.

Supplementary Fig. 8

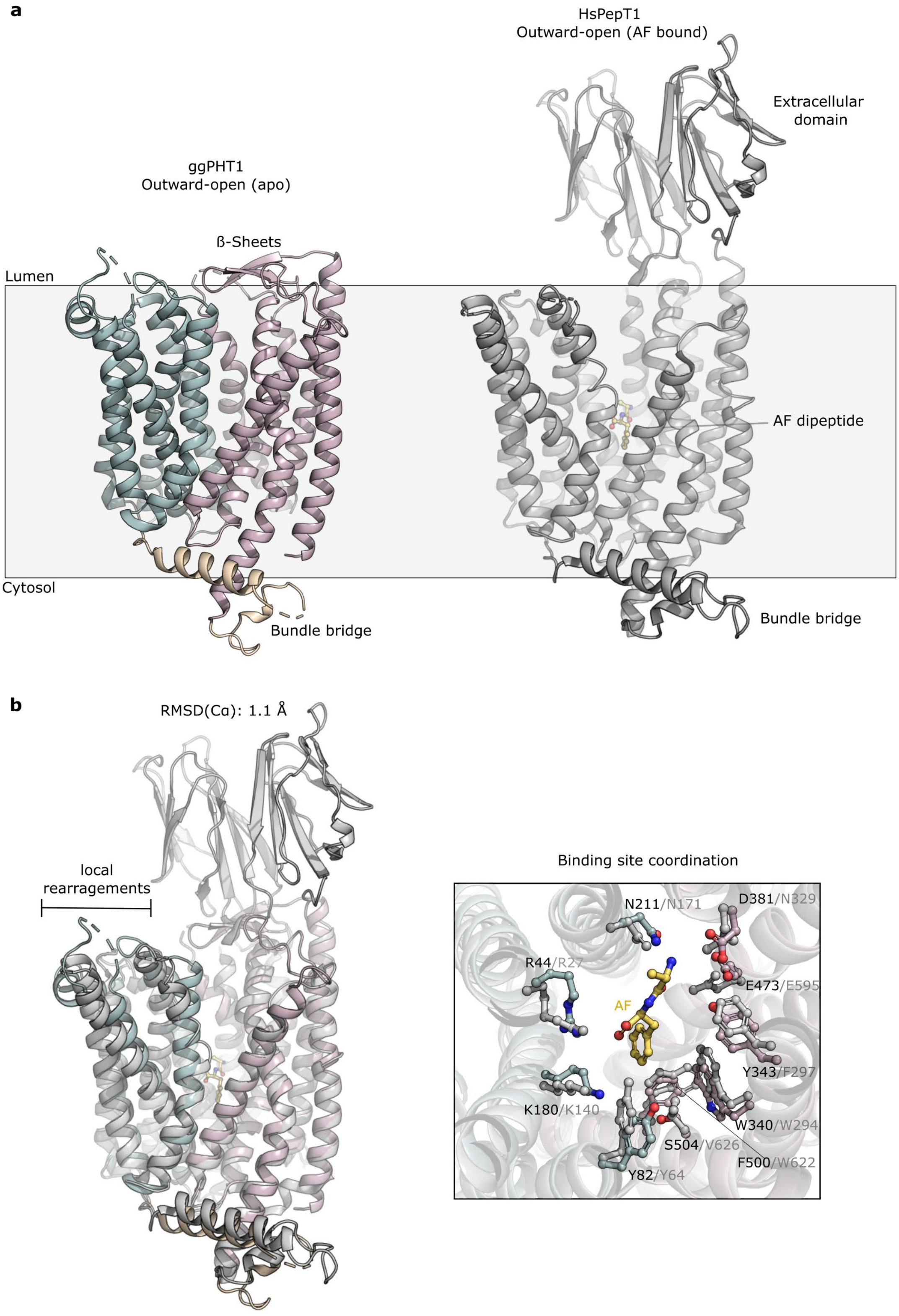

Supplementary Figure 8 – Structural comparison between the outward-open structures of PHT1 and PepT1. a) Side-by-side comparison of the chicken PHT1 outward-open structure (this work) with the human PepT1 outward-open AF-bound structure (pdb 7PMX). b)

Superimposition of PHT1 and PepT1, relative to their C-domains, showing a similar binding site coordination.

Supplementary Fig. 9

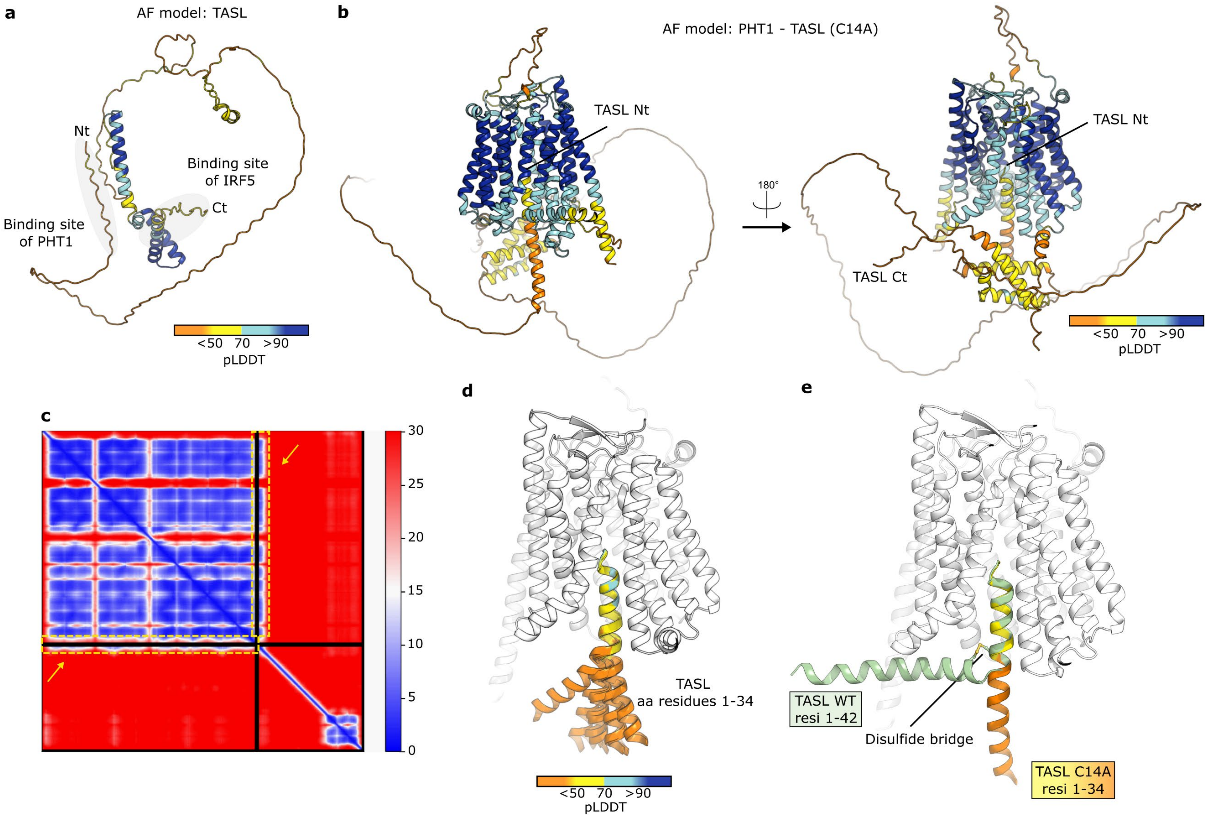

Supplementary Figure 9 - Estimated quality of structural models for the chicken PHT1-TASL complex, built using AlphaFold Multimer version 2.3.0. a) Cartoon view of the AF2 model of chicken TASL (Uniprot accession number A0A1L1RS25). TASL regions predicted to interacted with PHT1 or IRF5 are highlighted. b) Cartoon view of PHT1-TASL model colored by each residue's predicted local distance difference test (pLDDT) score. A residue with a pLDDT score that is greater than 90 (dark blue) indicates high estimated accuracy of the position of its backbone and sidechain rotamers. A pLDDT score above 70 (light blue) suggested that the prediction of the backbone is confident. c) Predicted alignment error (PAE) plot of the PHT1-TASL model shows the estimated expected distance error in Å. Each position (i,j) in the matrix is filled with the expected distance error in the residue i's position if the model and true (unknown) structure are aligned on residue j. The PAE scores are reported by AlphaFold and blue means low errors in this plot. The region of the N-terminus of TASL that binds to PHT1 is indicated with the yellow frame. Overall, the local structure of the interacting TASL N-terminus received only average pLDDT scores but the interaction region between the TASL N-terminus and PHT1 is predicted with high confidence by the PAE plots. d) Alignment of 10 best scoring models and close-up view of TASL (colored by pLDDT score) interaction with PHT1 (white). Disordered regions were hidden from this view. e) Overlay of wild-type

TASL (green) with TASL C14A (yellow-orange) complex models with PHT1. The non-conserved Cys14 residue of TASL has been replaced by an alanine during model predictions, as AlphaFold2 established a disulfide bridge between Cys14 and Cys20, unlikely to form given the reducing nature of the cytoplasm. The binding interface at the central cavity is identical in both models.

Supplementary Fig. 10

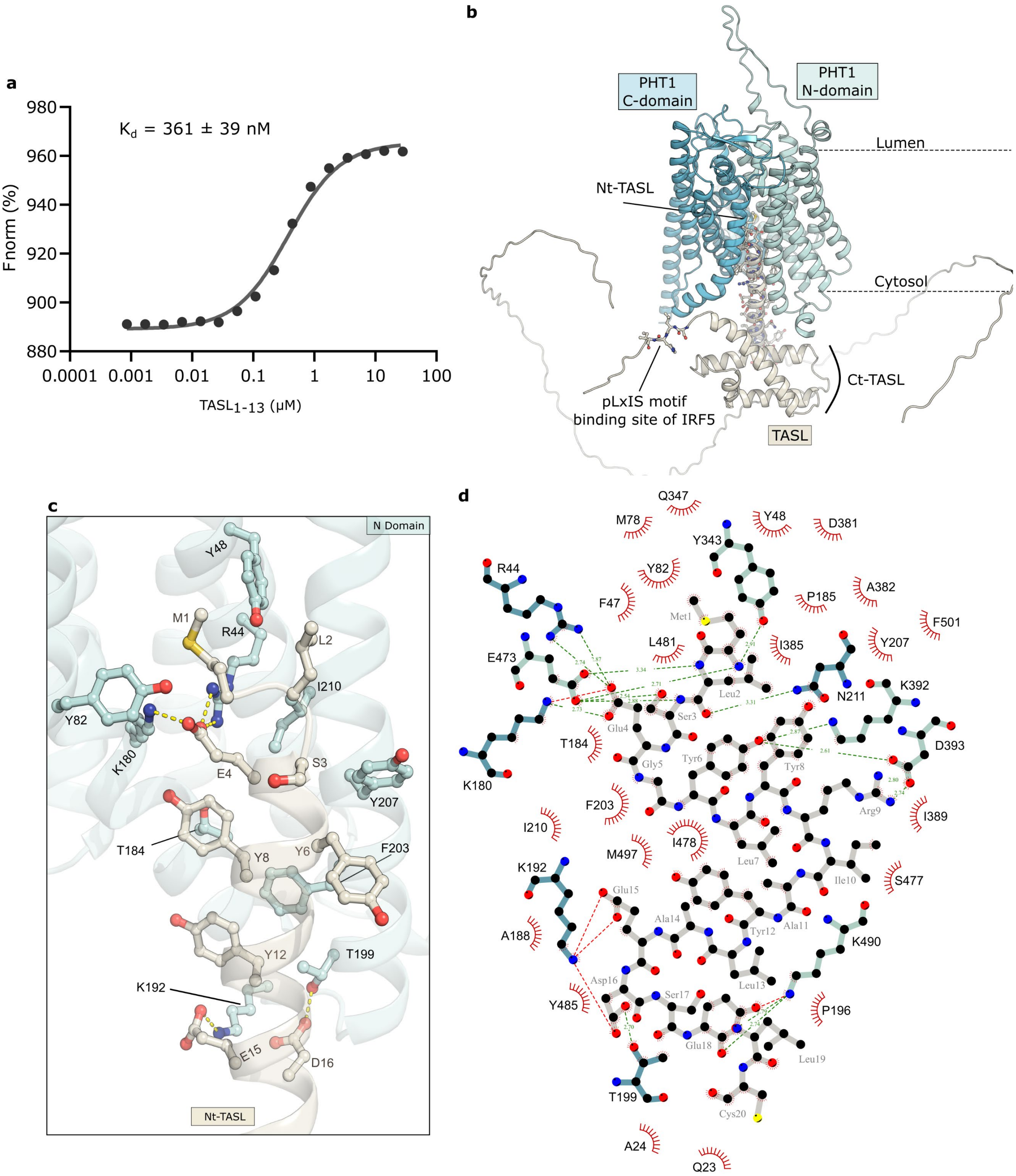

Supplementary Figure 10 – Binding and coordination between PHT1 and TASL. a) Microscale thermophoresis (MST) binding data of PHT1 with fluorescently labelled $TASL_{1-13}$ peptide. Affinity of PHT1 to TASL was approximately 360 nM, in good agreement with the affinity estimated by thermal unfolding assays. b) Cartoon representation of the AF2 model of PHT1 (blue) in complex with TASL (brown). The C-domain of TASL is shown and the pLxIS motif is shown as sticks. c) Close-up view of the N-domain interface of PHT1, in the AF2 model of PHT1-TASL. Selected residues are shown as sticks and yellow dashes indicate

hydrogen bonds (cut-off at 3.2 Å). d) Schematic generated by LigPlot$^+$ showing all the residues and contacts part of the PHT1-TASL interface. Green dashes highlight hydrogen bonds and corresponding length, red lines show salt-bridges, red spiked-semi-circles illustrate residues in PHT1 involved in hydrophobic contacts while the black circles with red spikes show the corresponding TASL residues involved in hydrophobic contacts. Source data for relevant information are provided as a Source Data file.

Supplementary Fig. 11

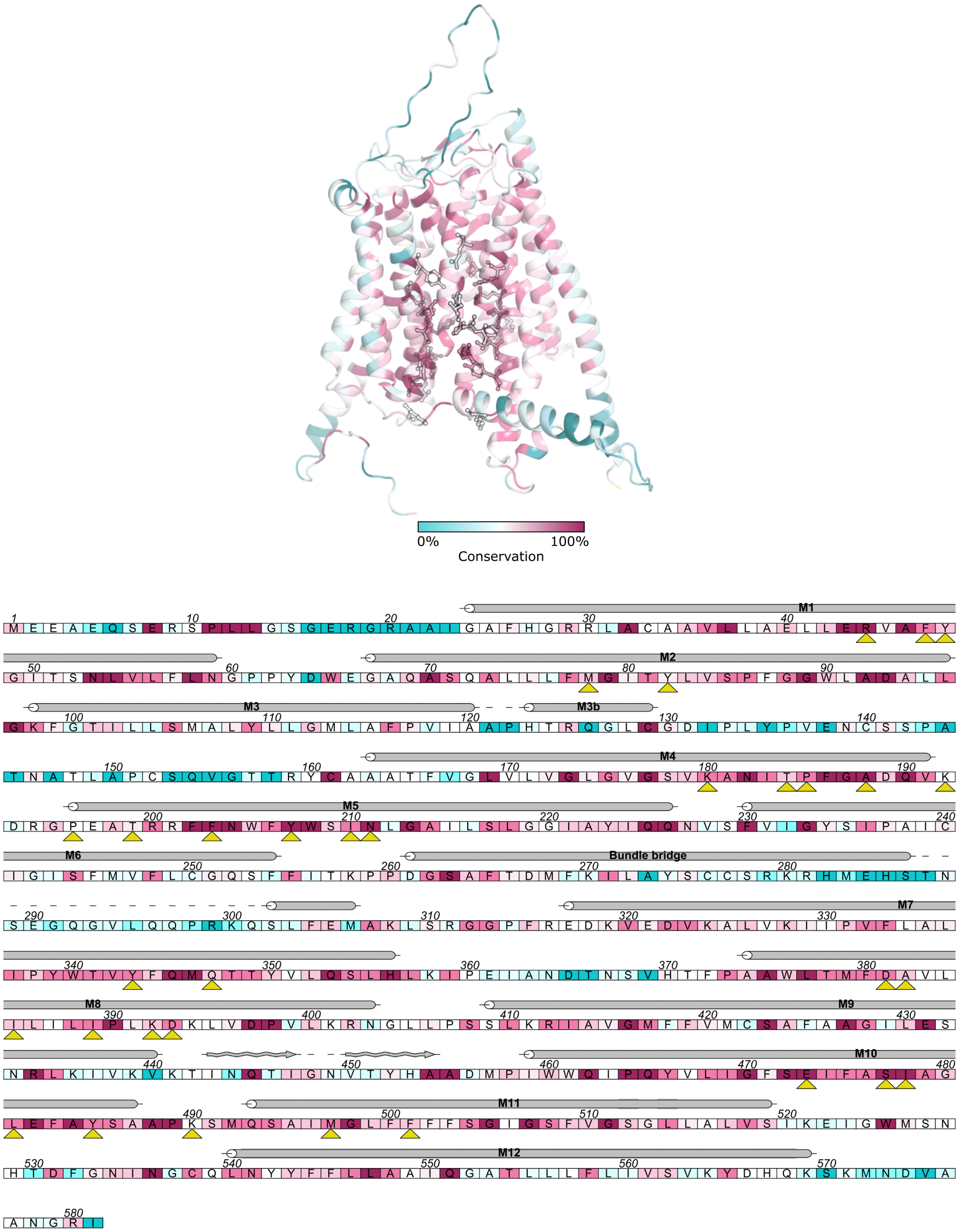

Supplementary Figure 11 – PHT1 sequence conservation. Cartoon representation of PHT1 and corresponding sequence are colored according to the conservation among 581 unique

mammalian sequences. Yellow triangles indicate residues that are part of the interface with TASL.

Supplementary Fig. 12

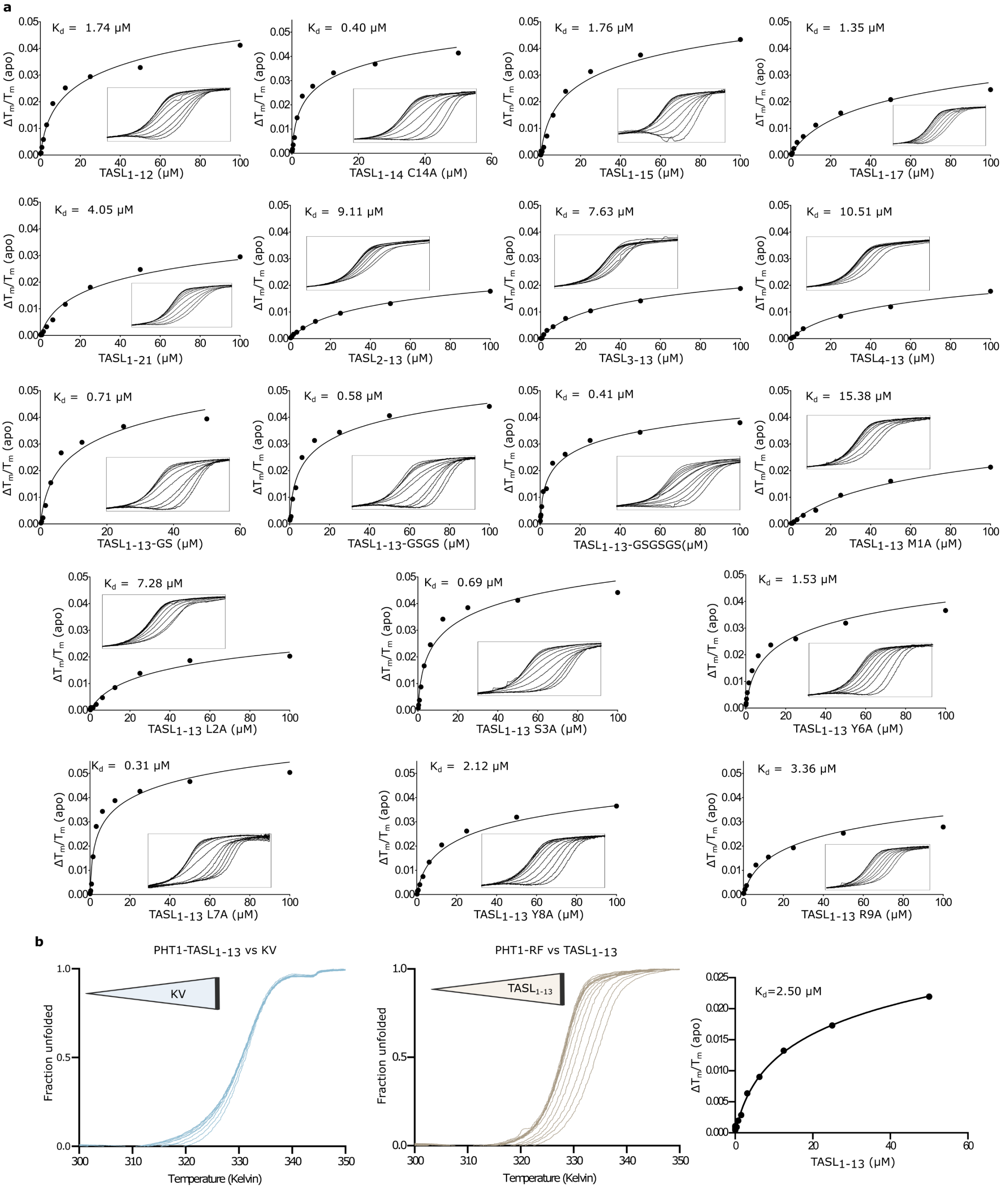

Supplementary Figure 12 - Thermal unfolding assays and affinities between PHT1 and TASL peptides. a) Thermal unfolding titration of different TASL peptides with PHT1. Data extrapolated from the F_{350} signal was fitted following a modified Hall's method and normalized unfolding transitions are shown. b) Competition assays between $TASL_{1-13}$ peptide and KV or RF dipeptide for PHT1 binding. PHT1 saturated with 10 μM of $TASL_{1-13}$ peptide was mixed

with 2-fold dilutions of KV dipeptide (highest concentration being 5 mM) and thermal unfolding of PHT1 was measured. The binding of KV dipeptide is blocked by the $TASL_{1-13}$ peptide (left). Competition assays between $TASL_{1-13}$ peptide and RF dipeptide for PHT1 binding. PHT1 saturated with 2 mM RF dipeptide was mixed with 2-fold dilutions of $TASL_{1-13}$ peptide (highest concentration being 50 µM) and thermal unfolding of PHT1 was measured. The presence of RF dipeptide reduces significantly the affinity of $TASL_{1-13}$ peptide towards PHT1. Data extrapolated from the $F_{350:330}$ signal were fitted following a modified Hall's approach (right). Source data for relevant information are provided in the Source Data file.

Supplementary Fig. 13

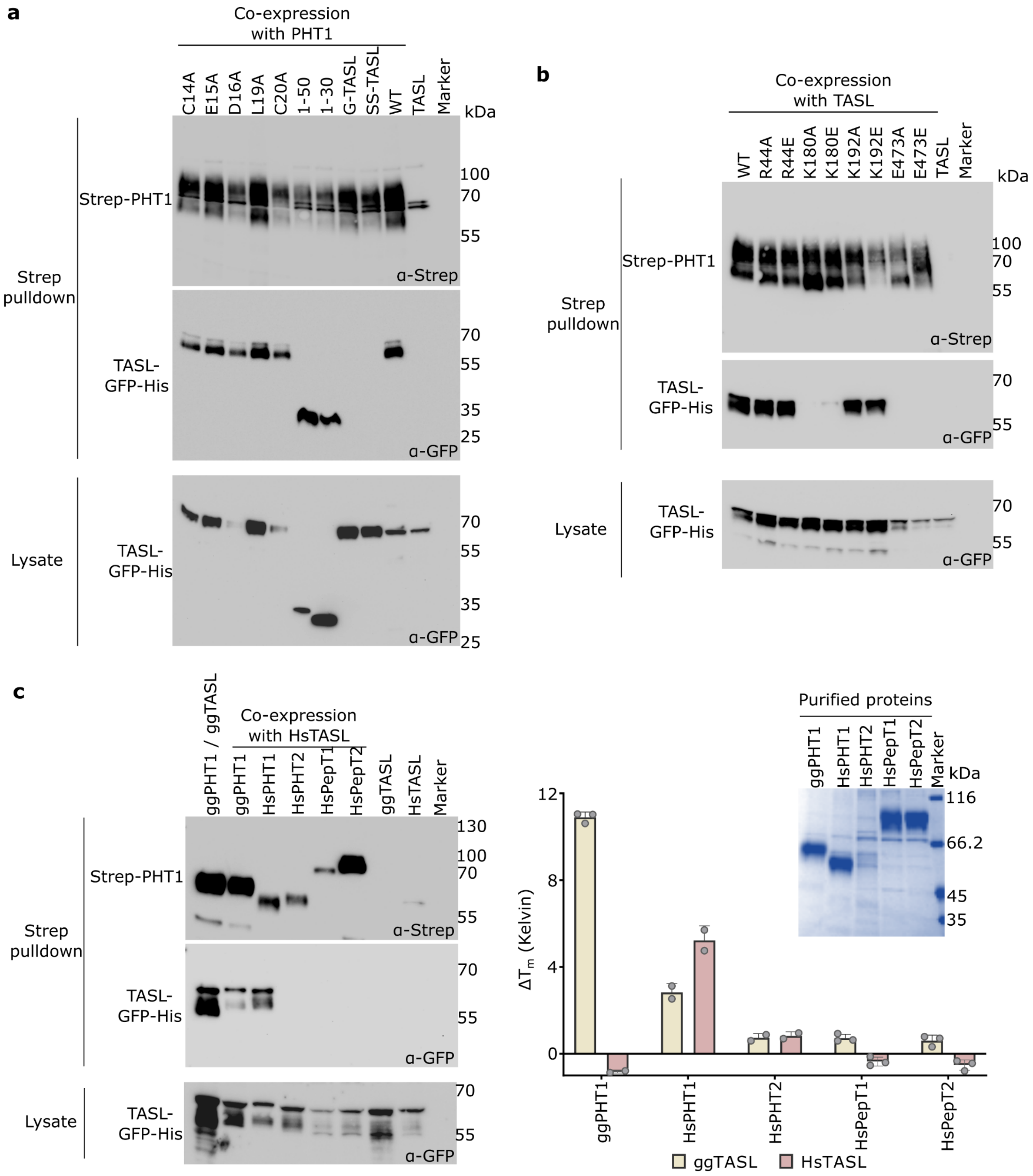

Supplementary Figure 13 – Pull-down assays of PHT1 with TASL mutants and control proteins. a) Pull-down assay using Twin-Streptavidin-tagged PHT1 with GFP-His-tagged TASL mutants at the interface residues. Elute and lysate samples were analyzed by western-blot. Data shown are representative of two independent experiments with similar results. b) Pull-down assay using Twin-Streptavidin-tagged PHT1 mutants at the interface residues with GFP-His-tagged TASL. Elute and lysate samples were analyzed by western-blot. Data shown are representative of two independent experiments with similar results. c) Pull-down assay using Twin-Streptavidin-tagged transporter proteins and GFP-His-tagged TASL (left). Thermal stability of transporter proteins screened with chicken or human TASL peptides

(residues 1-13) at a concentration of 50 μM. Changes in the melting temperature of the transporter protein in the presence of a peptide as compared to the melting temperature of the protein alone (ΔT_m) are shown. Data represent the mean ± SD of two or more technical replicates (right). Source data for relevant information are provided as a Source Data file.

Supplementary Table 1 – Sequences of the unique sybodies identified by ELISA

Sybody	Sequence
1	QVQLVESGGGLVQAGGSLRLSCAASGFPVYSRHMYWYRQAPGKEREWVAAIASQGMKTWYADSVKGRFTISRDNAKNTVYLQMNSLKPEDTAVYYCNVKDTGQWWGLYDYWGQGTQVTVSAGRAGEQKLISEEDLNSAVDHHHHHH
2	QVQLVESGGGLVQAGGSLRLSCAASGFPVNTRTMHWYRQAPGKEREWVAAIYSSGQNTFYADSVKGRFTISRDNAKNTVYLQMNSLKPEDTAVYYCNVKDWGNIWEYDYWGQGTQVTVSAGRAGEQKLISEEDLNSAVDHHHHHH
3	QVQLVESGGGLVQAGGSLRLSCAASGFPVWAAYMFWYRQAPGKEREWVAAIGSTGWYTRYADSVKGRFTISRDNAKNTVYLQMNSLKPEDTAVYYCHVKDAGWNWWAYDYWGQGTQVTVSAGRAGEQKLISEEDLNSAVDHHHHHH
6	QVQLVESGGGLVQAGGSLRLSCAASGFPVYATWMYWYRQAPGKEREWVAAISSTGWSTYYADSVKGRFTISRDNAKNTVYLQMNSLKPEDTAVYYCNVKDVGFWWDQYDYWGQGTQVTVSAGRAGEQKLISEEDLNSAVDHHHHHH
19	QVQLVESGGGSVQAGGSLRLSCAASGNIHAINYLGWFRQAPGKEREGVAALDTWVGYTYYADSVKGRFTVSLDNAKNTVYLQMNSLKPEDTALYYCAAAEWGWQGPLKMRAYEYWGQGTQVTVSAGRAGEQKLISEEDLNSAVDHHHHHH
20	QVQLVESGGGSVQAGGSLRLSCAASGNIWTIRYLGWFRQAPGKEREGVAALDTASGSTYYADSVKGRFTVSLDNAKNTVYLQMNSLKPEDTALYYCAAAWWGYTVPLMWQHYYYWGQGTQVTVSAGRAGEQKLISEEDLNSAVDHHHHHH
23	QVQLVESGGGSAQAGGSLRLSCAASGNIAHIYYLGWFRQAPGKEREGVAALMTFTGWTYYADSVKGRFTVSLDNAKNTVYLQMNSLKPEDTALYYCAAATHGNWNPLQSDRYQYWGQGTQVTVSAGRAGEQKLISEEDLNSAVDHHHHHH
24	QVQLVESGGGSVQAGGSLRLSCAASGAIERIWYLGWFRQAPGKEREGVAALITFTGHTYYADSVKGRFTVSLDNAKNTVYLQMNSLKPEDTALYYCAAAYDGWFSPLTTRRYRYWGQGTQVTVSAGRAGEQKLISEEDLNSAVDHHHHHH
25	QVQLVESGGGSVQAGGSLRLSCAASGNIWDIRYLGWFRQAPGKEREGVAALETQYGYTYYADSVKGRFTVSLDNAKNTVYLQMNSLKPEDTALYYCAAADIGYYTPLIHVGWYSYWGQGTQVTVSAGRAGEQKLISEEDLNSAVDHHHHHH
27	QVQLVESGGGSVQAGGSLRLSCAASGDIETIWYLGWFRQAPGKEREGVAALSTVTGSTYYADSVKGRFTVSLDNAKNTVYLQMNSLKPEDTALYYCAAAYTGWMAPLWQWVYSYWGQGTQVTVSAGRAGEQKLISEEDLNSAVDHHHHHH
30	QVQLVESGGGLVQAGGSLRLSCAASGFPVKSRRMYWYRQARGKEREWVAAIYSWGATTVYADSVKGRFTISRDNAKNTVYLQMNSLKPEDTAIYYCNVKDWGSWYQYYDYWGQGTQVTVSAGRAGEQKLISEEDLNSAVDHHHHHH
31	QVQLVESGGGLVQAGGSLRLSCAASGFPVSQNWMWWYRQAPGKEREWVAAISSKGQTTHYADSVKGRFTISRDNAKNTVYLQMNSLKPEDTAVYYCNVKDWGAYWYSYDYWGQGTQVTVSAGRAGEQKLISEEDLNSAVDHHHHHH
36	QVQLVESGGGLVQAGGSLRLSCAASGFPVKSRRMYWYRQARGKEREWVAAIYSWGATTVYADSVKGRFTISRDNAKNTVYLQMNSLKPEDTAIYYCNVKDWGSWYQYYDYWGQGTQVTVSAGRAGEQKLISEEDLNSAVDHHHHHH
38	QVQLVESGGGLVQAGGSLRLSCAASGFPVSQNWMWWYRQAPGKEREWVAAISSKGQTTHYADSVKGRFTISRDNAKNTVYLQMNSLKPEDTAVYYCNVKDWGAYWYSYDYWGQGTQVTVSAGRAGEQKLISEEDLNSAVDHHHHHH
44	QVQLVESGGGLVQAGGSLRLSCAASGFPVDATSMYWYRQAPGKEREWVAAINSTGWYTEYADSVKGRFTISRDNAKNTVYLQMNSLKPEDTAVYYCNVKDYGWYYWAYDYWGQGTQVTVSAGRAGEQKLISEEDLNSAVDHHHHHH
48	QVQLVESGGGLVQAGGSLRLSCAASGFPVRWHYMYWYRQAPGKEREWVAAISSHGTYTYYADSVKGRFTISRDNAKNTVYLQMNSLKPEDTAVYYCSVKDIGRWFSKYDYWGQGTQVTVSAGRAGEQKLISEEDLNSAVDHHHHHH

55	QVQLVESGGGLVQAGGSLRLSCAASGFPVAARWMRWYRQAPGKEREWVAAINSFGDYTEYADSVKGRFTISRDNAKNTVYLQMNSLKPEDTAVYYCNVKDYGSQWWAYDYWGQGTQVTVSAGRAGEQKLISEEDLNSAVDHHHHHH
57	QVQLVESGGGLVQAGGSLRLSCAASGFPVKQKWMRWYRQAPGKEREWVAAINSNGDYTEYADSVKGRFTISRDNAKNTVYLQMNSLKPEDTAVYYCNVRDYGDQLFEYDYWGQGTQVTVSAGRAGEQKLISEEDLNSAVDHHHHHH
58	QVQLVESGGGLVQAGGSLRLSCAASGFPVWTRWMRWYRQAPGKEREWVAAINSYGDYTEYADSVKGRFTISRDNAKNTVYLQMNSLKPEDTAVYYCNVRDNGAYWHYYDYWGQGTQVTVSAGRAGEQKLISEEDLNSAVDHHHHHH
66	QVQLVESGGGLVQAGGSLRLSCAASGFPVDTSYMYWYRQAPGKEREWVAAIWSWGQWTYYADSVKGRFTISRDNAKNTVYLQMNSLKPEDTAVYYCAVYVGQYYIGQGTQVTVSAGRAGEQKLISEEDLNSAVDHHHHHH
80	QVQLVESGGGSVQAGGSLRLSCAASGSISSITYLGWFRQAPGKEREGVAALFTWWGWTYYADSVKGRFTVSLDNAKNTVYLQMNSLKPEDTALYYCAAASEGMYWPLTKNHYSYWGQGTQVTVSAGRAGEQKLISEEDLNSAVDHHHHHH
87	QVQLVESGGGLVQAGGSLRLSCAASGFPVVHLSMAWYRQAPGKEREWVAAINSFGDHTHYADSVKGRFTISRDNAKNTVYLQMNSLKPEDTAVYYCNVKDSGWFWTQYDYWGQGTQVTVSAGRAGEQKLISEEDLNSAVDHHHHHH
88	QVQLVESGGGLVQAGGSLRLSCAASGFPVWHSYMYWYRQAPGKEREWVAAIGSTGWYTTYADSVKGRFTISRDNAKNTVYLQMNSLKPEDTAVYYCNVKDQGWIHKQYDYWGQGTQVTVSAGRAGEQKLISEEDLNSAVDHHHHHH
89	QVQLVESGGGLVQAGGSLRLSCAASGFSVNYSYMYWYRQAPGKEREWVAAISSWGWYTVYADSVKGRFTISRDNAKNTVYLQMNSLKPEDTAVYYCNVKDRGNDDSSYDYWGQGTQVTVSAGRAGEQKLISEEDLNSAVDHHHHHH
94	QVQLVESGGGLVQAGGSLRLSCAASGFPVYKASMYWYRQAPGKEREWVAAINSKGWWTEYADSVKGRFTISRDNAKNTVYLQMNSLKPEDTAVYYCNVKDYGYEQLWYDYWGQGTQVTVSAGRAGEQKLISEEDLNSAVDHHHHHH
100	QVQLVESGGGLVQAGGSLRLSCAASGFPVAQRVMHWYRQAPGKEREWVAAIHSAGAQTFYADSVKGRFTISRDNAKNTVYLQMNSLKPEDTAVYYCNVKDDGKYYWLYDYWGQGTQVTVSAGRAGEQKLISEEDLNSAVDHHHHHH
101	QVQLVESGGGLVQAGGSLRLSCAASGFPVWATYMYWYRQAPGKEREWVAAIGSTGWFTVYADSVKGRFTISRDNAKNTVYLQMNSLKPEDTAVYYCNVKDRGWQTAQYDYWGQGTQVTVSAGRAGEQKLISEEDLNSAVDHHHHHH
104	QVQLVESGGGLVQAGGSLRLSCAASGFPVWNSWMRWYRQAPGKEREWVAAINSYGDHTEYADSVKGRFTISRDNAKNTVYLQMNSLKPEDTAVYYCNVKDYGEWWNNYDYWGQGTQVTVSAGRAGEQKLISEEDLNSAVDHHHHHH
105	QVQLVESGGGLVQAGGSLRLSCAASGFPVYIKWMRWYRQAPGKEREWVAAISSNGEYTEYADSVKGRFTISRDNAKNTVYLQMNSLKPEDTAVYYCNVKDYGHTQWRYDYWGQGTQVTVSAGRAGEQKLISEEDLNSAVDHHHHHH
109	QVQLVESGGGLVQAGGSLRLSCAASGFPVGHKWMRWYRQAPGKEREWVAAINSHGEFTEYADSVKGRFTISRDNAKNTVYLQMNSLKPEDTAVYYCNVKDYGWTAWAYDYWGQGTQVTVSAGRAGEQKLISEEDLNSAVDHHHHHH
111	QVQLVESGGGLVQAGGSLRLSCAASGFPVYSSWMRWYRQAPGKEREWVAAINSHGDYTEYADSVKGRFTISRDNAKNTVYLQMNSLKPEDTAVYSCNVKDHGYYMKHYDYWGQGTQVTVSAGRAGEQKLISEEDLNSAVDHHHHHH
116	QVQLVESGGGPVQAGGSLRLSCAASGFPVNTRTMHWYRQAPGKEREWVAAIYSSGQNTFYADSVKGRFTISRDNAKNTVYLQMNSLKPEDTAVYYCNVKDWGNIWEYDYWGQGTQVTVSAGRAGEQKLISEEDLNSAVDHHHHHH
121	QVQLVESGGGLVQAGGSLRLSCAASGFPVEQSWMRWYRQAPGKEREWVAAINSWGEYTEYADSVKGRFTISRDNAKNTVYLQMNSLKPEDTAVYYCNVKDQGYEHQRYDYWGQGTQVTVSAGRAGEQKLISEEDLNSAVDHHHHHH

122	QVQLVESGGGSVQAGGSLRLSCAASGNIWTIRYLGWFRQAPGKEREGVAALDTASGSTYYADSVKGRFTVSLDNAKNTVYLQMNSLKPEDTALYYCAAAWWGYTVPLMWQHYYYWGQGTQVTVSAGRAGEQKLISEEDLNSAVDHHHHHH
134	QVQLVESGGGLVQAGGSLRLSCAASGFPVYQTYMYWYRQAPGKEREWVAAINSTGYFTRYADSVKGRFTISRDNAKNTVYLQMNSLKPEDTAVYYCNVKDLGWWSEQYDYWGQGTQVTVSAGRAGEQKLISEEDLNSAVDHHHHHH
139	QVQLVESGGGSVQAGGSLRLSCAASGHIRWIKYLGWFRQAPGKEREGVAALYTYEGQTYYADSVKGRFTVSLDNAKNTVYLQMNSLKPEDTALYYCAAAYFGAFYPLSYDAYYYWGQGTQVTVSAGRAGEQKLISEEDLNSAVDHHHHHH
140	QVQLVESGGGLVQAGGSLRLSCAASGFPVWATYMYWYRQAPGKEREWVAAIGSTGWFTVYADSVKGRFTISRDNAKNTVYLQMNSLKPEDTAVYYCNVKDYGWFEYRYDYWGQGTQVTVSAGRAGEQKLISEEDLNSAVDHHHHHH
143	QVQLVESGGGLVQAGGSLRLSCAASGFPVYSAVMYWYRQAPGKEREWVASIGSTGQHTYYADSVKGRFTISRDNAKNTVYLQMNSLKPEDTAVYYCNVKDVGEYMYWYDYWGQGTQVTVSAGRAGEQKLISEEDLNSAVDHHHHHH
146	QVQLVESGGGLVQAGGSLRLSCAASGFPVDTTNMHWYRQAPGKEREWVAAIHSSGTYTFYADSVKGRFTISRDNAKNTVYLQMNSLKPEDTAVYYCNVKDKGAHLWWYDYWGQGTQVTVSAGRAGEQKLISEEDLNSAVDHHHHHH
155	QVQLVESGGGLVQAGGSLRLSCAAGGFPVKTSWMRWYRQAPGKEREWVAAINSTGDWTEYADSVKGRFTISRDNAKNTVYLQMNSLKPEDTAVYYCNVKDYGVQTYYYDYWGQGTQVTVSAGRAGEQKLISEEDLNSAVDHHHHHH

Supplementary Table 2 - Cryo-EM data collection, refinement and validation statistics

**Protein reconstructed**	PHT1-Sb27
**PDB accession code**	NA
**EMDB accession code**	NA

**Data acquisition**	
Microscope/Detector	Titan Krios/Gatan K3
Imaging software	EPU
Magnification	105,000
Voltage (kV)	300
Electron exposure (e-/Å^2)	75
Dose rate (e-/pix/s)	19.5
Frame exposure (e-/Å^2)	1.5
Defocus range (µm)	-0.9 to -2
Physical pixel size (Å)	0.85
Micrographs	53,070

**Reconstruction**	
Picked coordinates (cryolo)	7,347,786
Particles in 3D classification (RELION)	6,644,417
Particles in final refinement (CryoSPARC)	1,328,233
Symmetry imposed	C1
Map sharpening method for final atomic model refinement	CryoSPARCv3
Map sharpening method for illustration (trained model)	DeepEMhancer (tight target)
Map resolution, $FSC_{half\ maps;\ 0.143}$ masked/unmasked (Å)	3.3/3.4

**Refinement**	
Initial model used for refinement	AlphaFold2 model, relaxed with Amber
Model resolution (Å)	
FSC 0.143, masked/unmasked	3.3/3.3
Model composition	
Atoms (Hydrogens)	11,397 (5,679)
Protein residues	742
ADP B factor (Å^2) mean	79.17
R.m.s deviations	
Bond lengths (Å) (#>4σ)	0.005 (0)
Bond angles (°) (#>4σ)	0.480 (0)
Validation	
MolProbity score	1.59
Clashscore	6.38
Rotamer outliers (%)	0.67
Ramachandran plot	
Favored (%)	96.43
Allowed (%)	3.57
Outliers (%)	0.00

Supplementary Table 3 - PHT1-TASL model quality assessments and interaction interface properties calculated by AlphaPulldown package. iPTM and iPTM+pTM scores are reported by AlphaFold. Both scores range between 0 and 1 with 1 being the best. The remaining measurements in the table were done after removing disordered regions from TASL model, which correspond to all the residues after residue 43, to reflect the properties of the interface involving only the N-terminus of TASL. pDockQ score is calculated using the formula given by [2]. pDockQ evaluates the quality of the interface and it ranges between 0.018 and 0.742 and a higher pDockQ score indicates better quality at the interface. The rest of the rows are evaluations reported by PI-score pipeline [3]. PI-score is a binary classifier that will assign a class label to a protein complex structure. A positive PI-score classifies the complex as a "native-like" fit whereas a negative PI-score indicates otherwise. Num_intf_residues: number of residues at the interface. Polar: number of polar residues (Ser, Thr, Asn, Gln, His and Tyr) at the interface. Hydrophobic: number of hydrophobic residues (Ala, Leu, Ile, Val, Phe, Trp, Cys, Met) at the interface. Charged: number of charged residues (Asp, Glu, Lys, Arg) at the interface. contact_pairs: number of atomic contacts between the interface residues. sc: geometric shape complementarity of protein-protein interfaces. sc ranges between 0 and 1 with an sc score of 1, two proteins mesh precisely. hb: number of hydrogen-bonds in the interface. sb: number of salt-bridges at the interface. int_solv_en: interface solvation energy. int_area: interface surface area that will be inaccessible to solvent upon the interface formation. In summary, the model obtained moderate AlphaFold fold scores and PI-score classifies the interface as a true interface.

Num_intf_residues	16
Polar	0.188
Hydrophobhic	0.563
Charged	0.125
contact_pairs	12
sc	0.65
hb	15
sb	13
int_solv_en	-16.25
int_area	1289.45
pi_score	1.34
iptm_ptm	0.670
iptm	0.681
pDockQ	0.189

Supplementary Table 4 – TASL peptide sequences and determined affinities. The amino acid sequences of the TASL peptides used in this study are described below. Affinities between TASL peptides and PHT1 were determined by thermal shift assays and are summarized in the table below.

	Peptide sequence	K_D (μM)
Peptide lengths		
TASL $_{1-7}$	MLSEGYL	ND
TASL $_{1-9}$	MLSEGYLYR	ND
TASL $_{1-11}$	MLSEGYLYRIA	ND
TASL $_{1-12}$	MLSEGYLYRIAY	1.74 ± 0.33
TASL $_{1-13}$	MLSEGYLYRIAYL	0.36 ± 0.079
TASL $_{1-15}$	MLSEGYLYRIAYLCE	1.76 ± 0.32
TASL $_{1-17}$	MLSEGYLYRIAYLCEDS	1.35 ± 0.19
TASL $_{1-21}$	MLSEGYLYRIAYLCEDSELCT	4.05 ± 2.65
Deletions/ insertions		
TASL $_{2-13}$	LSEGYLYRIAYL	9.11 ± 1.11
TASL $_{3-13}$	SEGYLYRIAYL	7.63 ± 0.92
TASL $_{4-13}$	EGYLYRIAYL	10.51 ± 1.35
TASL $_{5-13}$	GYLYRIAYL	ND
G-TASL $_{1-13}$	GMLSEGYLYRIAYL	ND
SS-TASL $_{1-13}$	SSMLSEGYLYRIAYL	ND
TASL $_{1-13}$-GS	MLSEGYLYRIAYLGS	0.71 ± 0.05
TASL $_{1-13}$-GSGS	MLSEGYLYRIAYLGSGS	0.58 ± 0.04
TASL $_{1-13}$-GSGSGS	MLSEGYLYRIAYLGSGSGS	0.41 ± 0.06
Mutations		
TASL $_{1-13}$ M1A	ALSEGYLYRIAYL	15.38 ± 2.67
TASL $_{1-13}$ L2A	MASEGYLYRIAYL	7.28 ± 2.68
TASL $_{1-13}$ S3A	MLAEGYLYRIAYL	0.69 ± 0.26
TASL $_{1-13}$ E4A	MLSAGYLYRIAYL	ND
TASL $_{1-13}$ Y6A	MLSEGALYRIAYL	1.53 ± 0.57
TASL $_{1-13}$ L7A	MLSEGYAYRIAYL	0.31 ± 0.11
TASL $_{1-13}$ Y8A	MLSEGYLARIAYL	2.12 ± 0.73
TASL $_{1-13}$ R9A	MLSEGYLYAIAYL	3.36 ± 1.06
TASL $_{1-14}$ C14A	MLSEGYLYRIAYLA	0.40 ± 0.12

*ND – Not determined since no thermal induced shift at the concentration of 50 μM was observed.

Supplementary References

1. Heinz, L. X. *et al.* TASL is the SLC15A4-associated adaptor for IRF5 activation by TLR7–9. *Nature* **581**, 316–322 (2020).
2. Bryant, P., Pozzati, G. & Elofsson, A. Improved prediction of protein-protein interactions using AlphaFold2. *Nat. Commun.* **13**, 1265 (2022).
3. Malhotra, S., Joseph, A. P., Thiyagalingam, J. & Topf, M. Assessment of protein–protein interfaces in cryo-EM derived assemblies. *Nat. Commun.* **12**, 3399 (2021).
