# Supplementary material for: Molecular basis of TASL recruitment by the peptide/histidine transporter 1, PHT1
Source: Nat Commun. 2023 Sep 14;14:5696. doi: 10.1038/s41467-023-41420-5 (PMC10502012; doi:10.1038/s41467-023-41420-5)
Supplement: Supplementary file 3 — Reporting Summary [file 41467_2023_41420_MOESM3_ESM.pdf]

nature portfolio

Corresponding author(s): Christian Loew

Last updated by author(s): Aug 24, 2023

Reporting Summary

Nature Portfolio wishes to improve the reproducibility of the work that we publish. This form provides structure for consistency and transparency in reporting. For further information on Nature Portfolio policies, see our Editorial Policies and the Editorial Policy Checklist.

Statistics

For all statistical analyses, confirm that the following items are present in the figure legend, table legend, main text, or Methods section.

n/a	Confirmed	
☐	☒	The exact sample size (*n*) for each experimental group/condition, given as a discrete number and unit of measurement
☐	☒	A statement on whether measurements were taken from distinct samples or whether the same sample was measured repeatedly
☒	☐	The statistical test(s) used AND whether they are one- or two-sided<br>*Only common tests should be described solely by name; describe more complex techniques in the Methods section.*
☒	☐	A description of all covariates tested
☒	☐	A description of any assumptions or corrections, such as tests of normality and adjustment for multiple comparisons
☐	☒	A full description of the statistical parameters including central tendency (e.g. means) or other basic estimates (e.g. regression coefficient) AND variation (e.g. standard deviation) or associated estimates of uncertainty (e.g. confidence intervals)
☒	☐	For null hypothesis testing, the test statistic (e.g. *F*, *t*, *r*) with confidence intervals, effect sizes, degrees of freedom and *P* value noted<br>*Give P values as exact values whenever suitable.*
☒	☐	For Bayesian analysis, information on the choice of priors and Markov chain Monte Carlo settings
☒	☐	For hierarchical and complex designs, identification of the appropriate level for tests and full reporting of outcomes
☒	☐	Estimates of effect sizes (e.g. Cohen's *d*, Pearson's *r*), indicating how they were calculated

Our web collection on statistics for biologists contains articles on many of the points above.

Software and code

Policy information about availability of computer code

Data collection	The Cryo-EM data was collected using EPU2.8.0.1256REL (Thermo Fisher Scientific);<br>The Thermal Stability data was collected using PR. ThermControl (version 2.1.2);<br>Biolayer interferometry data was collected using the Octet System Data Acquisition v11.0 (FortéBio)
Data analysis	The Cryo-EM data was processed using Relion-3.1 and CryoSPARCv3. raw movies were motion-corrected using MotionCor2 in Relion.<br>Refinement and validation: Phenix 1.20.1; Coot 0.9.8.1; ChimeraX 1.3; PyMOL v1.5.0.4; MolProbity 4.2<br>For sequence alignments PROMAlS3D (no version) and visualization ALINE 1.0.025; ConSurf (no version); phylogenetic tree constructed in MEGA v10.1.8<br>Structure modeling was performed using AlphaPulldown;<br>Thermal Stability data was analyzed using MoltenProt v0.2.1; Visualized using Prism 9.5.1<br>Biolayer interferometry data was analyzed using the Octet Data Analy9.5.1sis software v10.0 (FortéBio)

For manuscripts utilizing custom algorithms or software that are central to the research but not yet described in published literature, software must be made available to editors and reviewers. We strongly encourage code deposition in a community repository (e.g. GitHub). See the Nature Portfolio guidelines for submitting code & software for further information.

Data

Policy information about availability of data

All manuscripts must include a data availability statement. This statement should provide the following information, where applicable:

- Accession codes, unique identifiers, or web links for publicly available datasets
- A description of any restrictions on data availability
- For clinical datasets or third party data, please ensure that the statement adheres to our policy

The EM data and fitted models for chicken PHT1 have been deposited in the Electron Microscopy Data Bank under accession code EMD-16758 [https://www.ebi.ac.uk/pdbe/entry/emdb/EMD-16758] and the PDB under accession code 8CNI [http://doi.org/10.2210/pdb8CNI/pdb]. The outward-open structure of human PepT1, used for comparative analysis in this study, can be found in the PDB under accession code 7PMX [http://doi.org/10.2210/pdb7PMX/pdb]. The AlphaFold model of the complex has been deposited in the ModelArchive [https://www.modelarchive.org/doi/10.5452/ma-oj2xo]. All protein sequences used in this study are publicly available at Uniprot (https://www.uniprot.org/) with the following accession codes: chicken PHT1 (Uniprot accession number F1NG54), human PHT1 (accession number Q8N697), PHT2 (accession number Q8IY34), chicken TASL (Uniprot accession number A0A1L1RS25), human TASL (Uniprot accession number Q9HAI6), human SLC15A5 (accession number A6NIM6), human Pept1 (accession number P46059) and human PepT2 (accession number Q16348). Source data are provided with this paper.

Human research participants

Policy information about studies involving human research participants and Sex and Gender in Research.

Reporting on sex and gender	*Use the terms sex (biological attribute) and gender (shaped by social and cultural circumstances) carefully in order to avoid confusing both terms. Indicate if findings apply to only one sex or gender; describe whether sex and gender were considered in study design whether sex and/or gender was determined based on self-reporting or assigned and methods used. Provide in the source data disaggregated sex and gender data where this information has been collected, and consent has been obtained for sharing of individual-level data; provide overall numbers in this Reporting Summary. Please state if this information has not been collected. Report sex- and gender-based analyses where performed, justify reasons for lack of sex- and gender-based analysis.*
Population characteristics	*Describe the covariate-relevant population characteristics of the human research participants (e.g. age, genotypic information, past and current diagnosis and treatment categories). If you filled out the behavioural & social sciences study design questions and have nothing to add here, write "See above."*
Recruitment	*Describe how participants were recruited. Outline any potential self-selection bias or other biases that may be present and how these are likely to impact results.*
Ethics oversight	*Identify the organization(s) that approved the study protocol.*

Note that full information on the approval of the study protocol must also be provided in the manuscript.

Field-specific reporting

Please select the one below that is the best fit for your research. If you are not sure, read the appropriate sections before making your selection.

☒ Life sciences ☐ Behavioural & social sciences ☐ Ecological, evolutionary & environmental sciences

For a reference copy of the document with all sections, see nature.com/documents/nr-reporting-summary-flat.pdf

Life sciences study design

All studies must disclose on these points even when the disclosure is negative.

Sample size	Biophysical assays were typically performed at least in triplicate (n=3) (unless otherwise stated), to ascertain accurate values of the data shown. No sample-size calculation was performed but statistical methods were used to calculate standard deviation as noted in figure legends.
Data exclusions	No data were excluded.
Replication	Thermal shift data were repeated at least once (except for concentration dependent assays of TASL peptide mutants) and it was reproducible. MST data were repeated at least twice and it was reproducible. Pull down assays were repeated at least once and it was reproducible. BLI assays were repeated at least once and it was reproducible.
Randomization	Samples were not randomized for this study as this is not applicable to the study.
Blinding	Blinding was not perform as is not applicable to the study.

Reporting for specific materials, systems and methods

We require information from authors about some types of materials, experimental systems and methods used in many studies. Here, indicate whether each material, system or method listed is relevant to your study. If you are not sure if a list item applies to your research, read the appropriate section before selecting a response.

Materials & experimental systems

n/a	Involved in the study
☐	☒ Antibodies
☐	☒ Eukaryotic cell lines
☒	☐ Palaeontology and archaeology
☒	☐ Animals and other organisms
☒	☐ Clinical data
☒	☐ Dual use research of concern

Methods

n/a	Involved in the study
☒	☐ ChIP-seq
☒	☐ Flow cytometry
☒	☐ MRI-based neuroimaging

Antibodies

Antibodies used	Mouse monoclonal anti myc (Sigma, Cat.#M4439, Batch #0000129827, dilution 1:2000)<br>Mouse monoclonal anti GFP, HRP conjugated (Miltenyi Biotec, Cat #130-091-833, Lot #523020687, dilution 1:4000)
Validation	The anti myc antibody was used for ELISA and the anti GFP-HRP antibody was used for western blot. The antibodies were bought from commercial vendors and validation for applications can be found on the manufacturers website.

Eukaryotic cell lines

Policy information about cell lines and Sex and Gender in Research

Cell line source(s)	Expi293F™ (Cat.#100044202) and Expi293F™ GnTI- (Cat.# A39240) were purchased from ThermoFisher
Authentication	No authentication of the cell line was done by the authors.
Mycoplasma contamination	We confirmed that all cell lines tested negative for mycoplasma contamination.
Commonly misidentified lines<br>(See ICLAC register)	No commonly misidentified cell lines were used in this study.
